# Outcomes and complications of cataract surgery in patients with chronic ocular graft-versus-host-disease—a multicenter, retrospective analysis

**DOI:** 10.1007/s00417-022-05613-w

**Published:** 2022-03-07

**Authors:** Uta Gehlsen, Christiane Faust, Christiane Blecha, Tina Dietrich-Ntoukas, Philipp Eberwein, Susanne Issleib, Tobias Meyer-ter-Vehn, Regine Braun, Henrike Westekemper, Philipp Steven

**Affiliations:** 1 Division for Dry-Eye and Ocular GvHD, Department of Ophthalmology, Medical Faculty, University and University Hospital of Cologne, Kerpener Strasse 62, 50937 Cologne, Germany; 2 Cluster of Excellence: Cellular Stress Responses in Ageing-Associated Diseases, CECAD, University of Cologne, Cologne, Germany; 3 Department of Ophthalmology, University Hospital Regensburg, Regensburg, Germany; 4 Department of Ophthalmology, Charité – Universitaetsmedizin Berlin, Corporate Member of Freie Universitaet Berlin, Humboldt Universitaet Berlin, and Berlin Institute of Health, Berlin, Germany; 5 Eye Center, University Medical Center Freiburg, Freiburg, Germany; 6 AugenCentrum Rosenheim, Rosenheim, Germany; 7 University Eye Hospital, University Wuerzburg, Wuerzburg, Germany; 8 Department of Ophthalmology, University Hospital Essen, University Duisburg Essen, Essen, Germany

**Keywords:** Graft-versus-host-disease, Cataract, Phacoemulsification, Complication

## Abstract

**Purpose:**

To evaluate the outcome of phacoemulsification in patients with chronic ocular Graft-versus-host disease (oGVHD) after allogeneic hematopoietic stem cell transplantation (aHSCT).

**Methods:**

Retrospective, observational multicenter study from 1507 oGVHD patients. From the patient files, data were collected including best-corrected visual acuity (BCVA), intraocular pressure (IOP), Schirmer’s test *I*, tear film break-up time (TFBUT), corneal fluorescein staining score, postoperative complications, and pre- and post-operative topical therapy.

**Results:**

Seventy-three patients underwent cataract surgery in 104 eyes. In *n* = 84 eyes, the oGVHD NIH grade was documented; 12% (*n* = 12) of analyzed eyes were staged oGVHD NIH grade 1, 31% (*n* = 32) NIH 2 and 39% (*n* = 41) NIH 3. The mean BCVA improved in 82% of the eyes (*n* = 86 eyes). BCVA significantly increased from 0.7 ± 0.5 to 0.4 ± 0.4 LogMAR after surgery independent from oGVHD severity. The mean IOP decreased from 14 ± 4 to 13 ± 4 mmHg after surgery. Visual acuity was moderately correlated to the pre-operative degree of corneal staining (Pearson *p* = 0.26, *p* = 0.002, Cohen’s effect size *f* = 0.29). The visual acuity decreased by 0.078 LogMar units (95% CI = 0.027–0.141) with each increase of corneal staining by one grade (*p* = 0.05). After surgery, corneal epitheliopathy increased significantly in 42% (*n* = 44) of the eyes. Postoperative complications included corneal perforation (*n* = 6, 6%), cystoid macular edema (*n* = 4, 4%), and endophthalmitis (*n* = 1, 1%).

**Conclusion:**

Phacoemulsification in patients with chronic oGVHD significantly improves visual acuity, but is associated with an increased risk of complications in particular corneal epitheliopathy and corneal perforations.

**Supplementary Information:**

The online version contains supplementary material available at 10.1007/s00417-022-05613-w.


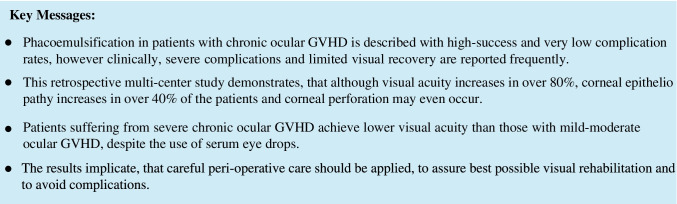


## Introduction

Chronic graft-versus-host-disease (GVHD) is a major cause of morbidity in patients after allogeneic hematopoietic stem cell transplantation (aHSCT) as treatment for several hematological disorders such as acute lymphoblastic or myeloid leukemia. Acute or chronic ocular GvHD (oGVHD) is described in up to 60% of the patients, mainly affecting the ocular surface and adnexa [1–4]. Resulting in disturbed tear production [5], loss of meibomian glands [6–8], conjunctival scarring [9], corneal epithelial defects and perforation [10], corneal and conjunctival ulceration [11, 12], inflammation as well as consecutive ocular discomfort, pain and blurred vision, oGVHD leads to a significant reduction in the vision-related quality of life in these patients [13, 14]. As increasing numbers of patients are receiving aHSCT and transplantation techniques and conditioning strategies have improved, patients’ survival rates also increase [15]. Consequently, the number of oGVHD patients also increases with a consecutive increase of long-term complications.

One of the most common non-GVHD ocular pathologies following aHSCT is the formation of mainly subcapsular cataracts. Cataracts are caused by exposure to total body irradiation and/or chemotherapy, high-dose systemic corticosteroids, and also the prolonged use of topical or high-dose systemic corticosteroids for prevention or treatment of systemic acute or chronic GvHD [16–18]. Due to significant visual impairment related to cataracts, many patients subsequently have to undergo phacoemulsification.

Generally, cataract surgery is a safe and effective procedure to restore visual acuity with very low complication rates [19, 20]. In oGVHD patients, phacoemulsification is also considered safe without increased likelihood of severe complications [21–25], although post-operative findings are described such as aggravation of ocular surface disease including filamentary keratitis, corneal ulceration, and perforation. Other, presumably non-oGVHD related postoperative complications are cystoid macular edema (CME) and posterior capsular opacification [14, 22]. Publications on outcome and complications of phacoemulsification in oGVHD derive from single-center patient cohorts with low to moderate numbers (*n* = 7–41 patients [21, 22, 25]), and one larger study including *n* = 229 patients with *n* = 51 eyes undergoing cataract surgery [24]. Additionally, most studies published did not differentiate between severity of oGVHD. Shah et al. [23] evaluated the outcome in 10 eyes from 6 patients with severe GvHD and Saboo et al. [24] referred to two eyes treated with autologous serum eye drops as an indicator of severe ocular surface disease with distinct postoperative complications after cataract surgery. This published data, however, is often not reflected in daily clinical practice. Here, often patients with oGVHD that undergo cataract surgery present with severe worsening of the ocular surface and implicate a study with higher number of patients. Furthermore, neither the outcome of larger numbers of patients with severe oGVHD has been evaluated nor the outcome of phacoemulsification under the use of autologous serum tears.

The aim of this study was therefore to comparatively analyze data from 7 centers regarding the outcome after cataract surgery including complication rates in a larger number of patients, with a special focus on patients with severe oGvHD and application of autologous serum eye drops.

## Methods

### Patient demographics

For this retrospective, observational clinical study data from *n* = 1507 ocular GvHD patients from 7 centers were identified in local electronic medical softwares such as ORBIS® (Agfa HealthCare, Bonn, Germany) by using ICD codes (H58.21–3). Anonymized data from 104 eyes from 73 consecutive patients (*n* = 49 male, *n* = 24 female; average age 58 ± 11 years; range: 20–78 years) undergoing phacoemulsification between 05/2010 and 06/2018 were manually extracted and then submitted as Excel® files by the participating centers for further analysis. Thirty-five patients had bilateral surgery (48%). Cataract surgery was performed at a median interval of 3.4 ± 2.5 years (IQR: 2.3–4.8 years) after allogeneic hematopoietic stem cell transplantation (aHSCT).

Underlying hematological diseases, concomitant ophthalmological diseases and treatments are depicted in Supplemental Tables 1, 2, 3.

Best-corrected visual acuity (BCVA), intraocular pressure (IOP), Schirmer test *I* (without anesthesia), tear film break-up time (TFBUT), and corneal fluorescein staining score (Oxford Scale[26]) were acquired during clinical routine examinations before and between 7 days up to one year after surgery. The median duration of the follow-up examination was 64 ± 159 d (IQR: 28–145 days) after cataract surgery.

In this study, *n* = 53 (51%) of the patients were treated with undiluted autologous serum eye drops (ASED) produced in a sealed, sterile manufacturing system as described previously [27] or treated with albumin eye drops. In Germany, the application and coverage through public health insurance of ASED is restricted to most severely affected patients, based on decision by the Federal Constitutional Court in 2005. To qualify for cost-compensation and application of ASED, patients need to be threatened by “acute blindness” caused by corneal epitheliopathy. Although not evaluated in this survey, most patients that applied ASED (or albumin) were very likely to have been severely impacted by oGVHD before the first application of ASED. However, patients with severe corneal epitheliopathy not always received ASED, which may also depends on absent availabilities of this therapy, refusal of cost-compensation, inability to donate blood, etc. For further analysis, data were grouped in autologous serum (ASED; *n* = 43)/albumin eye drops (AED; *n* = 10)-treated eyes versus no ASED/AED-treated eyes. As inclusion criteria for further subgroup analysis regarding the impact of serum treatment, only eyes treated before and after surgery were included in the ASED/AED group (*n* = 38/8); eyes without ASED/AED were used as control group (*n* = 46) (Table 1). For further sub-analysis, eyes were grouped regarding their GvHD NIH severity grade (NIH 0–3) according to [2].

**Table 1:** Number of patients treated with autologous serum (ASED) or albumin eye drops (AED) before and after surgery in total and NIH 1, 2, and 3 group

	NIH1	NIH2	NIH3
	Number of patients (*n* =)/percentage	Number of patients (*n* =)/percentage	Number of patients (*n* =)/percentage
noASED/AED	10/91%	21/58%	15/33%
ASED	1/9%	9/25%	28/62%
AED	0/0%	6/17%	2/4%
Total number of patients	11	36	45

### Statistical analysis

For statistical analysis, data were tested for their normal distribution using Kolmogorov–Smirnov-test. Data were then analyzed using the non-parametric Wilcoxon signed-rank test for paired samples to compare data before and after surgery (SPSS software, version 25, IBM, Ehningen, Germany). P-values < 0.05 were considered to be significant. For comparison between ASED and NIH grade related groups Mann–Whitney-U Test or Kruskal Wallis test followed by a Bonferroni corrected Dunn’s post hoc analysis was used. All ophthalmological data are depicted as boxplot diagrams, demonstrating the median with 25% and 75% percentiles (box) as well as the highest and lowest values (whiskers). Outliers are depicted as circles. For correlation of visual outcome and ophthalmological parameters, the Spearman coefficient and a linear regression were calculated.

## Results

### Ophthalmological parameters

The mean best-corrected visual acuity (BCVA) in this study cohort improved significantly after surgery. The mean corneal staining significantly increased after surgery from 2.1 to 2.5 (Table 1). The mean intraocular pressure (IOP) decreased; Schirmer’s *I* value and tear film break-up time (TFBUT) were not influenced by cataract surgery. All parameters are described in detail in Table 2 (mean/median ± standard deviation (range: min–max)).

**Table 2:** Ophthalmological parameters of 104 eyes included in the analysis. After surgery visual acuity and intraocular pressure improved, but corneal staining exacerbated significantly. Schirmer’s and tear film break-up-time were not influenced. Data were presented as mean/median ± standard deviation (minimum–maximum value)

	Before surgery mean/median ± standard deviation (range)	After surgery mean/median ± standard deviation (range)	Significance
BCVA (LogMAR)	0.7/0.6 ± 0.5 (0.1–2.0)	0.4/0.2 ± 0.4 (0–2.0)	*p* = 0.0001
IOP (mmHg)	14/15 ± 4 (5–25)	13/12 ± 4 (5–23)	*p* = 0.0001
Staining (Oxford)	2.1/2 ± 1.4 (0–5)	2.5/2 ± 1.7 (0–5)	*p* = 0.009
Schirmer’s *I* (mm)	3/2 ± 6 (0–35)	4/1 ± 7 (0–35)	*p* = 0.6
TFBUT (s)	2.6/2 ± 2.2 (0–8)	2.5/2 ± 2 (0–8)	*p* = 0.4

### Impact of oGVHD severity on outcome of surgery

#### Visual function, intraocular pressure, tear film

Ocular GvHD severity before surgery was graded using NIH consensus criteria [2, 28, 29]. Twelve percent of the patients (*n* = 12 eyes) were classified as NIH grade 1, 31% (*n* = 32 eyes) NIH grade 2, and 39% (*n* = 41 eyes) were NIH grade 3. For 18% of the patients (*n* = 19 eyes), no information concerning the NIH grading was obtainable.

**Fig. 1:**
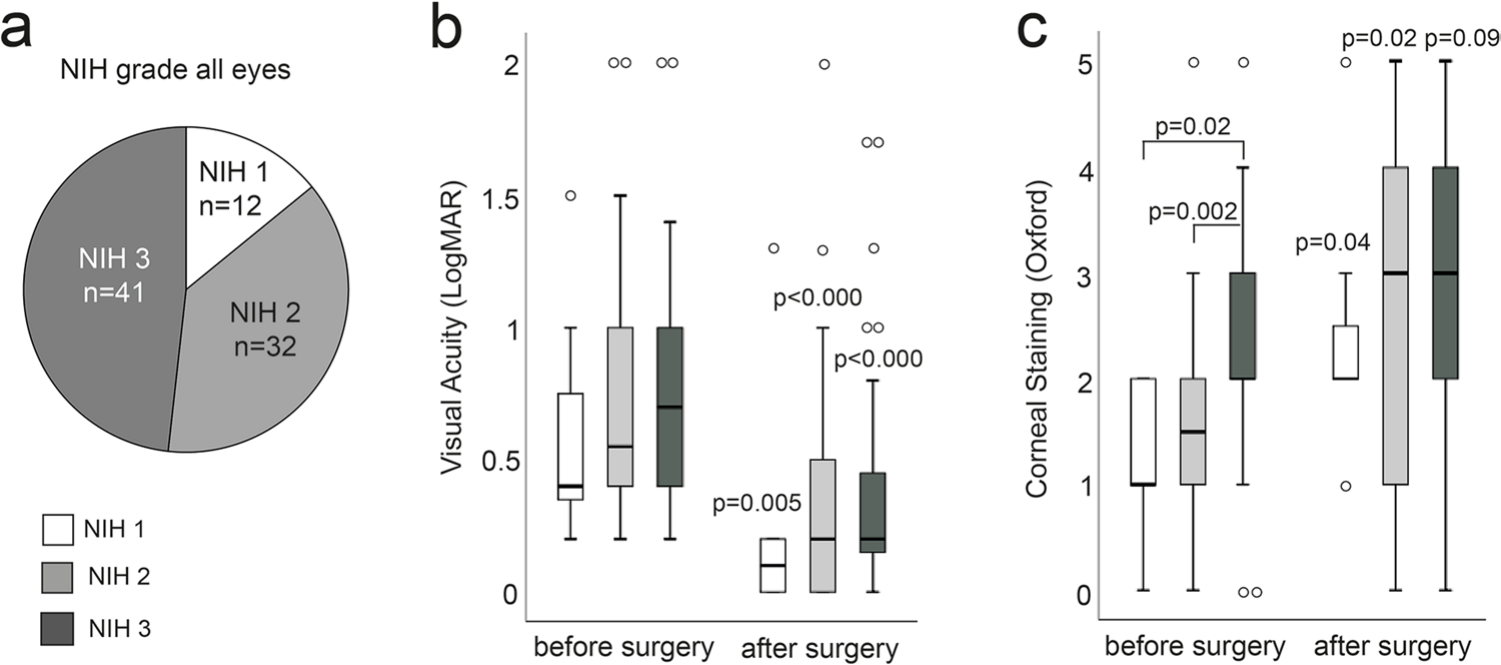
Outcome after cataract surgery related to severity of oGVHD (NIH grade 1–3). **a** NIH grade of *n* = 84 oGVHD eyes (in 20 eyes NIH grades were not documented). Twelve percent of analyzed eyes were graded NIH 1, 31% NIH 2, and 39% were NIH 3. **b** Visual acuity after cataract surgery improved in all groups independent from the NIH grade. **c** In NIH 1 and 2 staining increased after surgery to an NIH 3 level. NIH 3 group had a significantly higher corneal fluorescein staining at baseline compared to NIH 1 and 2

As a weak correlation between the NIH grade and the visual outcome after surgery was observed (Spearman-Rho coefficient: *ρ* = 0.2, *p* = 0.05), eyes were assigned to different groups according to the NIH grade and were compared regarding their visual outcome. The BCVA improved in all NIH groups (*p* = 0.0001), independent of the NIH grading: mean BVCA LogMAR before surgery was NIH 1: 0.6 ± 0.4, NIH 2: 0.7 ± 0.5, NIH 3: 0.8 ± 0.4 and mean BVCA LogMAR after surgery was NIH 1: 0.2 ± 0.4 (*p* = 0.005), NIH 2: 0.4 ± 0.5 (*p* < 0.001), NIH 3: 0.4 ± 0.4 (*p* < 0.001) (Fig. 1B). The overall success rate was 82%, with success defined as improvement of visual acuity after surgery (*n* = 85 eyes out of 104 eyes improved). In the NIH 1 group, success rate was 92% (*n* = 11 out of 12 eyes); in NIH 2 78% (*n* = 25 out of 32 eyes), and in NIH 3 82% (*n* = 34 out of 41 eyes). In eyes without NIH grade, available success rate was 79% (*n* = 15 out of 19 eyes).

All NIH groups demonstrated comparable values in Schirmer’s test and TFBUT before and after surgery although the TFBUT in NIH 3 was decreased compared to NIH 2 after surgery (*p* = 0.003). As expected after cataract surgery, the mean IOP values decreased in all eyes after surgery: NIH 1 (15 ± 4/13 ± 5 mmHG), NIH 2 (15 ± 3/13 ± 4 mmHg, *p* = 0.005), and NIH 3 (14 ± 4/12 ± 4 mmHg, *p* = 0.001). Detailed ophthalmological data are listed in Supplemental Table 4.

#### Corneal epitheliopathy

Since the postoperative visual outcome was correlated with the corneal staining before surgery (Spearman-Rho coefficient *ρ* = 0.5, *p* = 0.0001), a linear regression was calculated. The visual acuity was moderately correlated to the pre-operative degree of corneal staining (Pearson *r* = 0.26, *p* = 0.002, Cohen’s effect size *f* = 0.29). The visual acuity decreased by 0.078 LogMar units (95% CI = 0.027–0.141) with each increase of corneal staining by one grade (*p* = 0.05) (Supplemental Fig. 1). Due to the characteristics of the NIH criteria, a significant difference between the NIH groups existed before surgery (*p* = 0.003), with higher staining score in NIH 3 (mean ± SD: 2.8 ± 1.6) compared to NIH 1 (1.3 ± 0.7; *p* = 0.02) and NIH 2 (1.8 ± 0.4; *p* = 0.002). Detailed data are listed in Supplemental Table 4. After surgery, the staining increased significantly in NIH 2 (3.2 ± 0.4, *p* = 0.02) and NIH 1 (2.4 ± 1.2, *p* = 0.04) and reached a comparable degree of staining as in the NIH 3 (2.8 ± 1.6) group. The average staining in the NIH 3 group did not change after surgery (Fig. 1C).

**Fig. 2:**
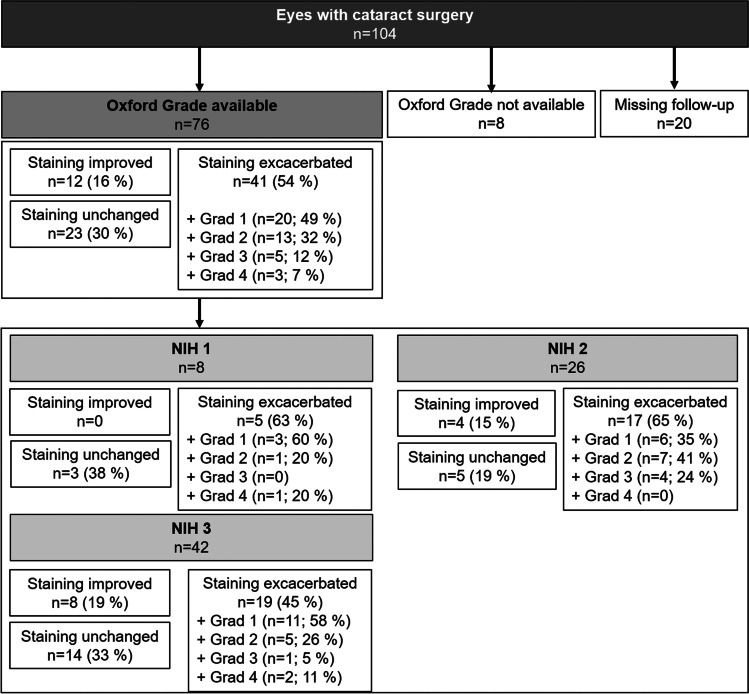
Corneal epitheliopathy after cataract surgery in *n* = 76 eyes calculated from the alteration in corneal staining (Oxford) before and after surgery. In 54% of the eyes, staining was impaired after surgery; in 23% of the eyes, corneal staining did not change. In 16%, corneal staining improved. In NIH 1 group, 63% of the eyes impaired, in NIH 2 65%, and in NIH 3 45%

Besides the increase of average staining after the procedure, exacerbation of corneal staining was documented in 46% (*n* = 48) of all 104 eyes investigated. The Oxford staining score was available in *n* = 76 eyes, in *n* = 8 eyes staining was not documented using the Oxford grading scale, and *n* = 20 data points were missing after surgery. In 49% (*n* = 20 eyes) out of these, the eyes worsened by 1 Oxford grade, 32% (*n* = 13) by 2 grades, 12% (*n* = 5) by 3 grades, and 7% (*n* = 3) by 4 grades. In 30% (*n* = 23) of all eyes, no change was documented; in 16% (*n* = 12) of the eyes, staining score improved after surgery (Fig. 2). The post-operative alteration of corneal staining did not correlate with NIH grade; in the NIH 1 group, 63% (*n* = 5) of the eyes worsened, in NIH 2 graded eyes 65% (*n* = 17), and in NIH 3 graded eyes 45% (*n* = 19) (Fig. 2).

#### ASED/AED treatment

The eyes were grouped in ASED/AED treated (*n* = 46) or control eyes (without ASED/AED treatment; *n* = 46) to analyze the impact of serum treatment. In both groups, visual acuity improved significantly after surgery (*p* = 0.0001 each). However, in eyes treated with ASED/AED, a significantly worse visual acuity was observed before and after surgery compared to control eyes (Table 2). ASED/AED eyes showed significantly higher staining before surgery compared to control eyes. After surgery, staining further increased in control eyes (*p* = 0.009) and by trend in ASED/AED eyes (*p* = 0.08) compared to pre-operative findings. After surgery staining in ASED/AED, the eyes also increased by trend (*p* = 0.06) compared to control eyes (Table 3). ASED/AED treatment did not affect IOP, TFBUT, or Schirmer’s in this study.

**Table 3:** Ophthalmological parameters before and outcome after cataract surgery in ASED/AED treated eyes compared to control eyes without ASED/AED

	ASED/AED		No ASED/AED	Significance (ASED/AED vs. control)
BCVA (LogMAR)	pre	0.8/0.7 ± 0.5 (0.2–2)	0.7/0.5 ± 0.4 (0.1–2)	*p* = 0.01
	post	0.4/0.2 ± 0.4 (0–2)	0.3/0.2 ± 0.4 (0–1.7)	*p* = 0.05
IOP (mmHg)	pre	14/14 ± 4 (5–20)	15/15 ± 4 (7–25)	*p* = 0.7
	post	12/12 ± 4 (5–23)	13/13 ± 4 (6–22)	*p* = 0.3
Staining (Oxford)	pre	2.6/3 ± 1.2 (1–5)	1.9/2 ± 1.4 (0–5)	*p* = 0.02
	post	3.1/3 ± 1.4 (1–5)	2.3/2 ± 2.7 (0–5)	*p* = 0.06
Schirmer’s *I* (mm)	pre	2/3 ± 3 (0–10)	4/1 ± 7 (0–35)	*p* = 0.9
	post	2/0 ± 3 (0–10)	6/3 ± 9 (0–35)	*p* = 0.4
TFBUT (s)	pre	2/1 ± 2 (0–8)	3/2 ± 2 (0–8)	*p* = 0.3
	post	2/1 ± 2 (0–8)	3/2 ± 2 (0–8)	*p* = 0.2

**Fig. 3:**
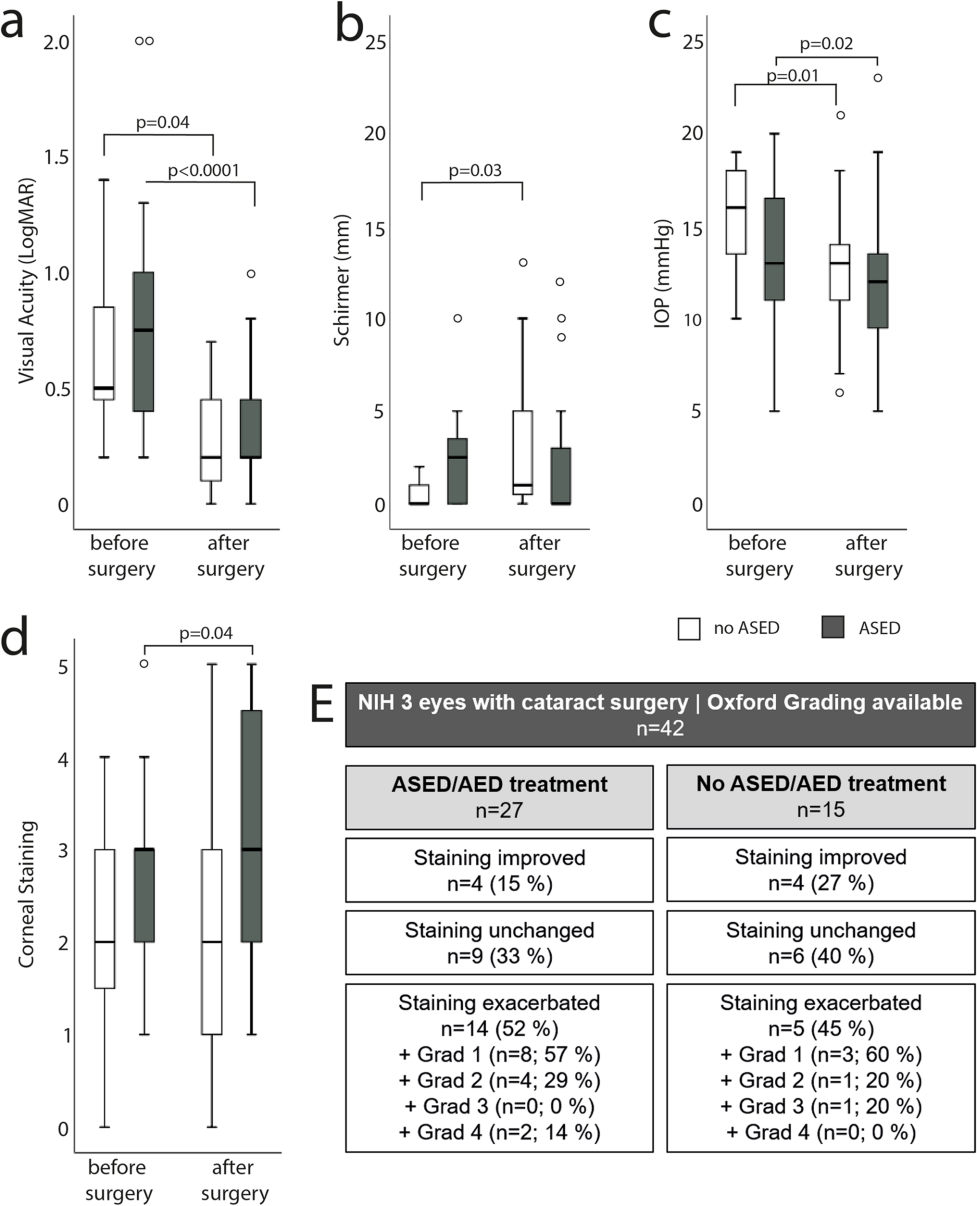
Ophthalmological parameters prior and after cataract surgery in *n* = 27 ASED-treated and *n* = 15 control eyes (no ASED) with NIH grade 3 only. **a** Visual acuity improved in both groups. **b** Schirmer’s increased in control eyes, but not in ASED-treated eyes. **c** Intraocular pressure decreased in both groups. **d** Mean corneal staining increased in ASED-treated eyes after surgery. **e** Changes in corneal epitheliopathy after surgery in ASED and control eyes. No statistical differences between both treatment groups were observed in the number of eyes exacerbating after surgery. (ASED = autologous serum eye drops)

Out of the NIH 1 group, 9% (*n* = 1) of the patients were treated with topical ASED or AED, 42% out of the NIH 2 (*n* = 9 ASED, *n* = 6 AED), but significantly more from the NIH 3 group (68%, *n* = 28 ASED, *n* = 2 AED) compared to NIH 2 (Table 1). To further investigate the influence of serum (ASED/AED) treatment on the outcome in severe cases of oGVHD, a sub-analysis of NIH 3 eyes was performed and ASED-treated (before and after surgery) eyes were compared with eyes without ASED (Fig. 3, Supplemental Table 5).

#### Complications

Increase in corneal staining was documented as the most common side effect after cataract surgery in 42 eyes (40% of all patients). Overall, 15 eyes, (14% of all patients) developed further complications after cataract surgery (Table 4). The second most frequent complication was corneal perforation in 6 eyes (6% of all eyes) and macular edema (CME) in 4 eyes (4% of all eyes). One patient developed (bacterial) endophthalmitis (1%). This patient later died due to severe complications of systemic GvHD.

**Table 4:** Cataract surgery-related post-operative complications. Overall *n* = 53 eyes (from 104 eyes overall) with complications were documented

Post-surgery findings/complications	*n* = (% of all eyes)
Increased corneal staining	44 (42%)
Corneal perforation	6 (6%)
Cystoid macular edema (CME)	4 (4%)
Corneal cloudiness	2 (2%)
Increase intraocular pressure	1 (1%)
Endophthalmitis	1 (1%)

Out of the *n* = 53 eyes with documented complications, 11% were NIH 1 (*n* = 6), 37% were NIH 2 (*n* = 20), and 36% NIH 3 (*n* = 19). In *n* = 8 eyes with complications, no NIH documentation was available.

In patients with ASED/AED treatment complications were documented in 56% (*n* = 26), and in patients without ASED/AED, 78% (*n* = 36) of the eyes developed complications. In 3 patients, a prolonged increased corneal staining occurred despite ASED treatment after cataract surgery. One patient reported worsening of epitheliopathy after discontinuation of ASED treatment. In severe NIH 3 graded eyes, the overall complication rate was 44% (*n* = 12) in the ASED/AED group, and 47% (*n* = 7) in the control group without ASED/AED. The number of severe complications in all eyes was decreased by trend (*p* = 0.09) in the ASED/AED group (6%, *n* = 1) compared to eyes without ASED/AED (27%, *n* = 9). In the NIH 3 subgroup, no severe complications were documented in the ASED/AED group, but two out of 7 eyes of the control group had severe complications after surgery. A statistical analysis was not applicable due to low numbers in the subgroups.

## Discussion

In concordance to other studies, postoperative visual improvement after phacoemulsification in ocular GVHD as the main outcome measure in this retrospective study was significant, resulting in a success rate of 82%. This is slightly lower than published outcomes of 87–97% [21, 23, 24] and possibly due to a larger proportion of cases with severe ocular GVHD in our cohort. In this context, the enhancement of visual acuity was correlated to the pre-operative degree of corneal staining but independent of the severity of oGVHD. A possible cause might be, that differences in NIH grading and in the availability of topical serum/albumin therapy in the contributing centers influenced the data or activity of ocular surface inflammation was higher than anticipated. It should be considered that sub-clinical ocular surface inflammation may have contributed to poor visual outcomes as discussed in [22]. Therefore, a revised grading scheme is warranted that enables better differentiation of oGVHD severity and activity in the future [30, 31].

It is well known that cataract surgery can induce or aggravate pre-existing dry eye disease, specifically corneal complications such as recurrent epithelial defects, filamentary, or punctate keratitis [32–34]. In this study, epitheliopathy aggravated in 42% and corneal staining increased significantly in the overall population investigated, independent from NIH grade. This increase could be due to desiccation during the procedure, epithelial toxicity of the disinfecting agents applied to the ocular surface, extend of the incisions, experience of the surgeon, time of the surgery and other factors that should be evaluated in a prospective follow-up study. Previously, the incidence of epithelial defects after cataract surgery in oGVHD was reported much lower between 0 and 8% [21, 24, 25]. Also, a temporary worsening of epithelial damages in 16% was reported 1–4 weeks after surgery [24], but the staining decreased postoperatively to a pre-surgery level after that time, comparable to patients without oGVHD or dry eye disease after cataract surgery [35]. However, in this study, corneal staining was still increased at up to 5 months after phacoemulsification. Six patients developed corneal erosions and five of these progressed to corneal perforations. Filamentary keratitis was present only in one patient but already diagnosed before surgery, which represents a lower rate than reported before [24].

Although controversially discussed, in our experience, ASED treatment using a sealed, sterile manufacturing system is an efficient and safe treatment option to improve visual outcome and ocular surface in chronic oGVHD [27]. Thirty-seven percent of the patients in this study were treated with 100% ASED, and an additional 8% with AED prior and after cataract surgery, however, that did not prevent increase of staining after phacoemulsification. An explanation could be, that ASED treatment was clinically only applied in severe cases, that otherwise would not have been suitable for cataract surgery, and that might have had a higher risk of corneal complications in general. However, although presenting with increased corneal staining, ASED-treated eyes demonstrated a lower rate of severe complications such as perforations. These findings support the strategy to pre-treat severe patients with serum eye drops to achieve a higher degree of peri-operative safety.

Elevated IOP is another general complication in oGVHD, due to the frequent use of steroids and corneal scarring [36]. Balaram et al. [21] reported elevated IOP after cataract surgery in three eyes as an early postoperative complication. In this study, intraocular pressure decreased slightly after surgery in 87% of the patients. However, the majority of the patients had an IOP ≤ 20 mmHg before and after surgery, which is within the normal range. Further severe complications in this study were CME in 4%, which was comparable to 4–5.6% as reported in [22, 24]. Posterior capsular opacification (PCO) requiring YAG capsulometry was documented in 2%, representing a distinct lower rate than published in [21] (62% PCO; 44% YAG) and [24] (18% PCO). However, the rate is likely to be much higher since the procedure is mainly performed in private practice and was therefore maybe not documented in the according files of the participating academic centers.

From our experience, phacoemulsification in oGVHD should be restricted to experienced surgeons. If cataract occurs following aHSCT, earlier rather than later surgery with respect to progression of oGVHD should also be considered in order to achieve better postoperative visual outcomes. However, in cases of highly active oGVHD associated with severe inflammatory activity on the ocular surface, cataract surgery may be postponed. If severe corneal epitheliopathy is present and surgery necessary, pre-operative application of autologous/allogeneic serum eye drops in addition to topical cyclosporine seems favorable. From these findings, the question arises, whether there is perioperative management that would further reduce complications and improve surgical outcome. Most participating centers now recommend a pre-operative application of topical corticosteroids (e.g. unpreserved dexamethasone TID for 3 consecutive days before surgery) followed by weekly examinations and aggressive therapy of ocular surface alterations as a standard. Overall, peri-operative care is crucial in oGVHD and external eye care providers should be informed about the increased complication risks in this special group of patients.

In summary, the frequent worsening of ocular surface disease after cataract surgery found in this study as a result of underlying oGVHD and/or as a consequence of the surgical procedure can cause severe complications which need to be monitored and treated. Future studies are warranted to establish guidelines on phacoemulsification in oGVHD, to enhance surgical outcome while reducing complications.

## Supplementary Information

Below is the link to the electronic supplementary material.

Supplementary file1 (DOCX 28 kb)
