# Supplementary material for: Outcomes and complications of cataract surgery in patients with chronic ocular graft-versus-host-disease—a multicenter, retrospective analysis
Source: Graefes Arch Clin Exp Ophthalmol. 2022 Mar 7;260(8):2613–22. doi: 10.1007/s00417-022-05613-w (PMC9325851; doi:10.1007/s00417-022-05613-w)
Supplement: Supplementary file 1 — Supplementary file1 (DOCX 28 kb) [file 417_2022_5613_MOESM1_ESM.docx]

**Supplemental data**

**Supplemental Figure 1:** Better visual outcome of cataract surgery was weakly correlated with lower corneal staining (p=0.0001; Spearman´s ρ=0.5) and NIH grade (p=0.02; Spearman´s ρ=0.25) prior to surgery.

**Supplemental Table 1:** Underlying diseases in 73 oGVHD patients included in the analysis.

| **Diagnosis** | **n=73 (%)** |
| --- | --- |
| Acute myeloid leukemia (AML) | 29 (40 %) |
| Acute lymphoblastic leukemia (ALL) | 5 (7 %) |
| Chronic lymphocytic leukemia (CLL) | 7 (10 %) |
| Chronic myelogenous leukemia (CML), Chronic myelomonocytic leukemia (CMML) | 5 (7 %), 1 (1 %) |
| Myelodysplastic syndrome/ myeloproliferative neoplasia (MDS) | 14 (19 %) |
| Multiple myeloma (MM) | 6 (8 %) |
| Other Non-Hodgkin‘s lymphoma (not specified) (NHL) | 6 (8 %) |

**Supplemental Table 2:** Pre-operative ophthalmological findings in 104 eyes included in the analysis (multiple findings/diagnoses are possible).

| **Preoperative ophthalmological findings** | **n=69** |
| --- | --- |
| Corneal scarring | 6 |
| Corneal erosio (perforating) | 4 (2) |
| Corneal vascularisation | 2 |
| Stromal calcification | 1 |
| Trichiasis | 4 |
| Amnion membrane transplantation | 7 |
| Perforating Keratoplasty | 1 |
| Keratitis filiformis | 1 |

**Supplemental Table 3:** Pre-operative concomitant systemic and topical therapy and treatments in 104 eyes included in the analysis (multiple treatments are possible).

| **Topical therapy** | **n=** |
| --- | --- |
| Artificial tears | 82 |
| Ointments (panthenol, vitamine A) | 31 |
| 0.05 % Cyclosporine A (CsA) | 62 |
| Tacrolimus, pimecrolimus | 11 |
| Autologous serum eye drops (ASED) | 46 |
| Albumin eye drops (AED) | 8 |
| Antibiotics | 3 |
| Glaucoma medication | 4 |
| Corticosteroids (prednisolone, dexamethasone) | 22 |
| Non-steroidal anti-inflammatory drug (NSAID) | 1 |
| Acetylcystein | 2 |
| **Ophthalmological treatments** |  |
| Punctum plugs | 4 |
| Lid margin hygiene | 3 |
| Therapeutic scleral contact lenses | 1 |
| **Systemic therapy** |  |
| Corticosteroids (prednisolone, prednison) | 20 |
| Immunosuppressives (mycophenolat-mofetil-MMF) | 10 |
| Cyclosporine A (CsA) | 6 |
| Pain reliever (metamizole, pregabalin) | 2 |
| Antibiotics | 2 |

**Suppl. Table 4:** Ophthalmological parameters of 104 eyes stratified into NIH grade 1-3. After surgery visual acuity in all groups, whereas intraocular pressure improved in NIH grade 2 and 3. Corneal staining exacerbates significantly in NIH grade 1 and 2. Schirmer´s and tear film break-up-time were not influenced. Data were presented as mean/median±standard deviation (minimum-maximum value).

| **NIH 1** | | | |
| --- | --- | --- | --- |
|  | **Before surgery**  mean/median±standard deviation (range) | **After surgery**  mean/median±standard deviation (range) | **Significance** |
| **BCVA (LogMAR)** | 0.6/0.4 ± 0.4 (0.2-1.5) | 0.2/0.1 ± 0.4 (0-1.3) | p=0.005 |
| **IOP (mmHg)** | 15/14 ± 5 (10-25) | 13/11 ± 5 (8-22) | p=0.4 |
| **Staining (Oxford)** | 1.3/1 ± 0.7 (0-2) | 2.4/2 ± 1.2 (1-5) | p=0.04 |
| **Schirmer´s I (mm)** | 4/3 ± 3 (0-10) | 5/4 ± 4 (0-10) | p=0.3 |
| **TFBUT (s)** | 3/2 ± 1.8 (1-7) | 2.5/2.5 ± 1 (1-4) | p=0.4 |

| **NIH 2** | | | |
| --- | --- | --- | --- |
|  | **Before surgery**  mean/median±standard deviation (range) | **After surgery**  mean/median±standard deviation (range) | **Significance** |
| **BCVA (LogMAR)** | 0.7/0.6 ± 0.5 (0.2-2.0) | 0.3/0.2 ± 0.5 (0-2.0) | p=0.0001 |
| **IOP (mmHg)** | 15/15 ± 3 (10-20) | 12/12 ± 3 (7-19) | p=0.005 |
| **Staining (Oxford)** | 1.5/1.5 ± 1.2 (0-5) | 2.6/3 ± 1.7 (0-5) | p=0.002 |
| **Schirmer´s I (mm)** | 6/3 ± 9 (0-35) | 8/3 ± 11 (0-35) | p=0.7 |
| **TFBUT (s)** | 3.6/2 ± 2.5 (1-8) | 2.6/3 ± 1.7 (0-5) | p=0.3 |

| **NIH 3** | | | |
| --- | --- | --- | --- |
|  | **Before surgery**  mean/median±standard deviation (range) | **After surgery**  mean/median±standard deviation (range) | **Significance** |
| **BCVA (LogMAR)** | 0.7/0.7 ± 0.4 (0.2-2.0) | 0.4/0.2 ± 0.4 (0-1.7) | p=0.0001 |
| **IOP (mmHg)** | 14/14 ± 4 (5-20) | 12/12 ± 4 (5-23) | p=0.0001 |
| **Staining (Oxford)** | 2.4/2 ± 1.2 (0-5) | 2.8/3 ± 1.5 (0-5) | p=0.09 |
| **Schirmer´s I (mm)** | 2.3/1 ± 3 (0-12) | 2.4/1 ± 3.5 (0-35) | p=0.9 |
| **TFBUT (s)** | 1.8/1 ± 2 (0-8) | 1.9/1 ± 2 (0-8) | p=0.7 |

**Suppl. Table 5:** Ophthalmological parameters and outcome after cataract surgery in NIH 3 eyes only, depending on topical treatment with or without ASED, AED. In NIH 3 eyes no treatment with AED was documented.

|  | | **ASED** | **No ASED** |
| --- | --- | --- | --- |
| **BCVA (LogMAR)** | pre | 0.7/0.8 ± 0.4 (0.2-1.3) | 0.8/0.7 ± 0.4 (0.2-1.3) |
|  | post | 0.4/0.2 ± 0.3 (0-1.3) | 0.3/0.3 ± 0.3 (0-1.3) |
| **IOP (mmHg)** | pre | 14/14 ± 4 (5-20) | 15/16 ± 3 (8-19) |
|  | post | 13/12 ± 4 (5-23) | 12/12 ± 4 (6-21) |
| **Corneal Staining (Oxford)** | pre | 2.4/2 ± 1.2 (1-5) | 2.2/2 ±1.4 (0-4) |
|  | post | 3.0/3 ± 1.3 (1-5) | 2.0/2.0 ± 1.7 (0-5) |
| **Schirmer´s I (mm)** | pre | 2/3 ± 3 (0-10) | 2/0 ± 4 (0-12) |
|  | post | 2/0 ± 3 (0-10) | 3/1 ± 4 (0-13) |
| **TFBUT (s)** | pre | 2/1 ± 2 (0-8) | 1/1 ± 2 (0-6) |
|  | post | 2/1 ± 2 (0-8) | 2/2 ± 2 (0-6) |
